# Testing the potential contribution of *Wolbachia* to speciation when cytoplasmic incompatibility becomes associated with host‐related reproductive isolation

**DOI:** 10.1111/mec.16157

**Published:** 2021-09-16

**Authors:** Daniel J. Bruzzese, Hannes Schuler, Thomas M. Wolfe, Mary M. Glover, Joseph V. Mastroni, Meredith M. Doellman, Cheyenne Tait, Wee L. Yee, Juan Rull, Martin Aluja, Glen Ray Hood, Robert B. Goughnour, Christian Stauffer, Patrik Nosil, Jeffery L. Feder

**Affiliations:** ^1^ Department of Biological Sciences University of Notre Dame Notre Dame IN USA; ^2^ Faculty of Science and Technology Free University of Bozen‐Bolzano Bozen‐Bolzano Italy; ^3^ Competence Centre for Plant Health Free University of Bozen‐Bolzano Bozen‐Bolzano Italy; ^4^ Department of Forest and Soil Sciences Boku, University of Natural Resources and Life Sciences Vienna Austria; ^5^ United States Department of Agriculture Temperate Tree Fruit & Vegetable Research Unit Agricultural Research Service Wapato WA USA; ^6^ Instituto de Ecología A.C. Xalapa México; ^7^ LIEMEN‐División Control Biológico de Plagas PROIMI Biotecnología‐CONICET Tucumán Argentina; ^8^ Department of Biological Sciences Wayne State University Detroit MI USA; ^9^ Washington State University Extension Vancouver WA USA; ^10^ CEFE University Montpellier CNRS EPHE IRD University Paul Valéry Montpellier 3 Montpellier France; ^11^ Department of Biology Utah State University UT USA

**Keywords:** cytoplasmic incompatibility, postmating isolation, premating isolation, *Rhagoletis cingulata*, *Rhagoletis indifferens*, *Wolbachia*

## Abstract

Endosymbiont‐induced cytoplasmic incompatibility (CI) may play an important role in arthropod speciation. However, whether CI consistently becomes associated or coupled with other host‐related forms of reproductive isolation (RI) to impede the transfer of endosymbionts between hybridizing populations and further the divergence process remains an open question. Here, we show that varying degrees of pre‐ and postmating RI exist among allopatric populations of two interbreeding cherry‐infesting tephritid fruit flies (*Rhagoletis cingulata* and *R*. *indifferens*) across North America. These flies display allochronic and sexual isolation among populations, as well as unidirectional reductions in egg hatch in hybrid crosses involving southwestern USA males. All populations are infected by a *Wolbachia* strain, *w*Cin2, whereas a second strain, *w*Cin3, only co‐infects flies from the southwest USA and Mexico. Strain *w*Cin3 is associated with a unique mitochondrial DNA haplotype and unidirectional postmating RI, implicating the strain as the cause of CI. When coupled with nonendosymbiont RI barriers, we estimate the strength of CI associated with *w*Cin3 would not prevent the strain from introgressing from infected southwestern to uninfected populations elsewhere in the USA if populations were to come into secondary contact and hybridize. In contrast, cytoplasmic–nuclear coupling may impede the transfer of *w*Cin3 if Mexican and USA populations were to come into contact. We discuss our results in the context of the general paucity of examples demonstrating stable *Wolbachia* hybrid zones and whether the spread of *Wolbachia* among taxa can be constrained in natural hybrid zones long enough for the endosymbiont to participate in speciation.

## 1 INTRODUCTION

New species of sexually reproducing organisms arise as heritable barriers to gene flow evolve between formerly interbreeding populations (Mayr, 1963). These barriers can take many different forms, causing populations to be both pre‐ and postzygotically reproductively isolated (Dobzhansky, 1982). Research on the causes of reproductive isolation (RI) has mainly focused on divergent ecological adaptation, sexual selection and intrinsic nuclear genomic incompatibilities (Coyne & Orr, 2004; Rundle & Nosil, 2005; Schluter, 2000). However, there is an increasing realization that intracellular endosymbionts may also contribute to RI between their hosts (Brucker & Bordenstein, 2012; Cooper et al., 2017; Coyne & Orr, 2004; Gebiola et al., 2016; Shropshire & Bordenstein, 2016; Werren, 1997). Maternally inherited endosymbionts, such as *Wolbachia* (Alphaproteobacteria) or *Cardinium* (Bacteroidetes) present in arthropods and nematodes, can cause cytoplasmic incompatibility (CI) (Engelstädter & Hurst, 2009; Perlman et al., 2014; Turelli, 1994; Werren et al., 2008). In such cases, crosses between males infected with the endosymbiont and uninfected females or females infected with a different endosymbiont strain result in embryonic death or inviability of offspring.

The transmission dynamics of CI‐inducing endosymbionts have raised several questions concerning their role in speciation (Brucker & Bordenstein, 2012; Werren, 1998). For example, if one allopatric population possesses *Wolbachia* and another does not, then CI is unidirectional. Following secondary contact, unidirectional CI can lead to the introgression of the endosymbiont into uninfected populations (Jiggins, 2003; Rousset & Solignac, 1995; Sanaei et al., 2021) if *Wolbachia* reaches a critical threshold frequency (Li & Wan, 2019; Souto‐Maior et al., 2015; Turelli, 1994). Once a *Wolbachia* strain becomes fixed in a population there would be no CI, as all matings would involve infected females and males (Hurst & Schilthuizen, 1998). Given these considerations, it has been argued that for endosymbionts to be involved in speciation it will probably require allopatric populations becoming fixed for different CI‐causing strains (Bordenstein et al., 2001; Brucker & Bordenstein, 2012; Telschow et al., 2005). In this case, bidirectional CI produces no or few viable embryos from hybrid crosses, which impedes the introgression of *Wolbachia* strains between populations.

However, there is no guarantee that strong bidirectional CI will persist. For example, CI between two populations can be eliminated if one population loses its endosymbiont due to inefficient vertical transmission (Engelstädter & Telschow, 2009; Frost et al., 2010; Hughes et al., 2014). Moreover, when CI is incomplete and a percentage of embryos survive (Vavre et al., 2002), the strain with the strongest CI phenotype or most efficient vertical transmission may displace the other, leading to its fixation (Kriesner et al., 2016). *Wolbachia* can also be transferred horizontally between hosts by third parties (e.g., via a predator–prey or parasite–host interaction), or through environmental contact (e.g., via a shared resource) (Baldo et al., 2008; Enigl & Schausberger, 2007; Le Clec’h et al., 2013; Morrow et al., 2014; Schuler et al., 2013; Tseng et al., 2020) or by hybridization with a closely related species (Rhitoban Raychoudhury et al., 2009; Sanaei et al., 2021; Turelli et al., 2018). Thus, for *Wolbachia* to play a role in speciation, CI must persist with the endosymbiont not spreading such that CI contributes to or allows for the accumulation of additional RI between diverging taxa. Understanding the role that endosymbionts may play in speciation therefore calls for more than just characterizing the agents and nature of CI between populations; it also requires us to determine the relationship and interaction of the CI caused by the endosymbiont(s) with other host‐related barriers to gene flow.

A key issue concerning the role that endosymbiont‐induced CI may play in speciation is thus the degree to which CI becomes coupled with other forms of nonendosymbiont RI to reduce gene flow between populations (Hurst & Schilthuizen, 1998; Shropshire & Bordenstein, 2016; Telschow et al., 2005; Werren, 1998). Coupling refers to when the effects of different barriers to gene flow become associated with one another such that their joint, cumulative effect can generate higher levels of RI than their individual effects alone (Barton, 1983; Butlin & Smadja, 2018; Flaxman et al., 2014; Nosil et al., 2021). In *Wolbachia*, nonendosymbiont‐related divergent ecological selection, sexual selection and nuclear encoded genetic incompatibilities may lower the level of effective gene flow between host populations below the critical migration rate necessary for *Wolbachia* to introgress and spread, resulting in the retention of CI (Flor et al., 2007; Telschow et al., 2002, 2005). As cytoplasmic and nuclear‐related forms of RI become associated in disequilibrium between host populations, the effect of their coupling on reducing gene flow can enable the evolution of additional RI (Shropshire & Bordenstein, 2016; Telschow et al., 2002, 2005). Indeed, the strongest empirical support for *Wolbachia* contributing to speciation may be found in systems where CI‐causing *Wolbachia* strains are associated with premating isolation or assortative mating (Gebiola et al., 2016; Miller et al., 2010; Shoemaker et al., 1999). Even if eventually lost, *Wolbachia* can still have played a role in speciation if the endosymbiont facilitated the evolution of non‐CI‐related host RI at some earlier stage of the divergence process. Thus, determining whether and how endosymbionts can couple with and facilitate the evolution of nonsymbiont‐based host RI barriers is central to understanding the role that CI may play in speciation.

**FIGURE 1:**
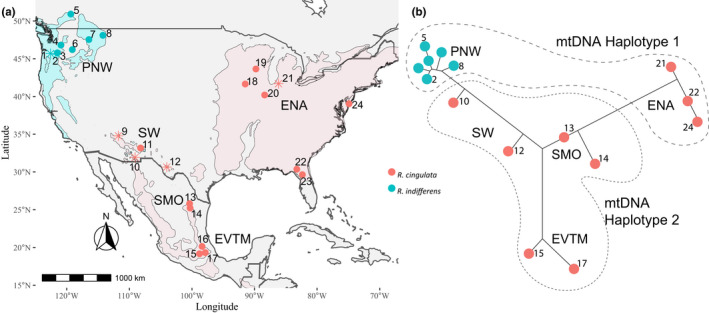
Collection sites for *Rhagoletis cingulata* and *R*. *indifferens* and their associated nuclear and mtDNA variation. (a) The ranges of cherry host plants, *Prunus serotina* and *P*. *emarginata*, are superimposed on the figure in pink and aqua, respectively. Sites 1–8 are *R*. *indifferens* populations in the Pacific Northwest (PNW), sites 9–12 are *R*. *cingulata* populations in the southwestern USA (SW), sites 13 and 14 are *R*. *cingulata* populations in the Sierra Madre Oriental Mountains of Mexico (SMO), sites 15–17 are *R*. *cingulata* populations in the central highlands of the Eje Volcánico Trans Mexicano of Mexico (EVTM), and sites 18–24 are *R*. *cingulata* populations in the eastern USA (ENA). Asterisks denote populations that were used in mating experiments. Locality information for each site is provided in Table S1. (b) Neighbour‐joining network for nuclear‐encoded microsatellites with superimposed mtDNA variation was modified with permission from Doellman et al. (2020). Nuclear markers show isolation‐by‐distance pattern of geographical variation that forms an arc from the ENA through the SW to the PNW and extends south latitudinally from the SW into the SMO and EVTM in Mexico. Dotted lines highlight the disjunct mtDNA haplotype distribution, with ENA and PNW populations sharing an mtDNA haplotype 1 that differs from the haplotype 2 possessed by SW and Mexican flies

The *Rhagoletis* (Diptera: Tephritidae) cherry fly system is an attractive model to test for possible *Wolbachia*‐induced CI and its relationship to other forms of pre‐ and postmating RI (Hood et al., 2012). *Rhagoletis cingulata* (Loew) and its primary host black cherry (*Prunus serotina*) are native to eastern North America (ENA), with geographically isolated populations residing on higher elevation mountaintops through the southwestern USA (SW) (Bush, 1966) (Figure 1a). Black cherry‐infesting populations are also found in two Mexican subpopulations: one in the Sierra Madre Oriental Mountains (SMO) and the other in the central highlands of the Eje Volcánico Trans Mexicano (EVTM) (Rull et al., 2011). *Rhagoletis indifferens* Curran and its primary host bitter cherry (*P*. *emarginata*) are present in the Pacific Northwest (PNW) and are separated from populations of ENA flies by the northern plains of North America and from SW flies by the Mojave Desert and Great Basin (Bush, 1966; Dowell & Penrose, 2012). The current biogeography of cherry flies in North America could be characterized as representing five regional populations (PNW, SW, SMO, EVTM and ENA) with the more diverged PNW population afforded species status (Bush, 1966; Doellman et al., 2019). However, whether *R*. *indifferens* in the PNW should be considered a separate species from *R*. *cingulata* as opposed to a genetically diverged population is an open question.

Previous studies have reported *Wolbachia* infections in *R*. *cingulata* populations in North America, as well as in its invasive range in Europe (Schuler et al., 2013; Wolfe et al., 2021). Postmating RI in the form of reduced egg hatch has been observed in crosses between Mexican and ENA flies (Tadeo et al., 2015). Moreover, distinct mitochondrial DNA (mtDNA) haplotypes were found in SW and Mexican vs. PNW and ENA populations (Doellman et al., 2019), which might indicate a *Wolbachia*‐driven selective sweep of a unique mtDNA haplotype (Hurst & Jiggins, 2005; Schuler et al., 2016). The disjunct distribution for mtDNA stands in contrast to an otherwise geographical pattern of isolation‐by‐distance for nuclear‐encoded microsatellites (Figure 1b; Doellman et al., 2019, 2020). Populations in the SW are estimated to have become isolated from those in ENA, the PNW and Mexico in the early Holocene 5500–8500 years ago (Doellman et al., 2020). At this time, climate change caused desertification of the region, fragmenting formerly contiguous forests into isolated mountaintop populations (Lomolino et al., 1989; Thompson & Anderson, 2000). The recent isolation of cherry fly populations means that any observed RI can be inferred to be relatively recent in origin as well, which is important for assessing the contribution of *Wolbachia* to population divergence.

To accomplish this goal, we first quantified levels of pre‐ and postmating RI that exist among cherry fly populations in the USA, including premating RI due to ecology (allochrony), sexual isolation (mate choice) and postmating incompatibilities (fecundity and egg hatch). Second, we assessed whether any postmating RI corresponds with the diverged mtDNA haplotype distinguishing SW and Mexican cherry flies from populations in the PNW and ENA (Doellman et al., 2019). Third, we genotyped *Wolbachia* from cherry fly populations across North America to assess whether different strains of *Wolbachia* are present and correspond to patterns of mtDNA divergence and postzygotic isolation detected between these flies. Together, these three aims allowed us to assess whether *Wolbachia*‐induced CI is coupled to other forms of RI that could prevent the introgression of the endosymbiont in the event of secondary contact and thus facilitate speciation.

## 2 METHODS

### 2.1 Insect collection and rearing

*Rhagoletis cingulata* and *R*. *indifferens* were collected as larvae from infested fruit at 24 sites from 2004 to 2018 across North America: including the SMO and EVTM in Mexico, and the PNW, SW and ENA in the USA and Canada (Figure 1; Table S1). Fly larvae were reared to adulthood following standard husbandry protocols (Feder et al., 1990; Tadeo et al., 2015; Yee, 2013), with Mexican samples reared at the Instituto de Ecología, Xalapa, Veracruz, and USA samples at the University of Notre Dame, Notre Dame, Indiana.

### 2.2 Allochronic isolation

The timing of adult eclosion is an important host‐related adaptation that generates ecologically based, allochronic premating isolation between *Rhagoletis* attacking different host plants (Feder & Filchak, 1999; Feder et al., 1997; Inskeep et al., 2021; Meyers et al., 2020). To evaluate allochronic isolation, we compared adult eclosion times for 939 flies collected in 2017 from PNW, SW and ENA populations. We recorded the number of days it took for pupae to eclose as adults when incubated at 24°C, 65% relative humidity and 14:10 h light–dark following a standardized 6‐month overwintering period at 4°C. Differences in eclosion times among populations were tested for significance by ANOVA (data were normally distributed) using population (PNW, SW, ENA) as the predictor variable and followed by Tukey honest significant difference (HSD) *post hoc* comparisons. Both were performed in R using the package stargazer (Hlavac, 2018; R Core Team, 2019). Pairwise estimates of allochronic isolation (AI) were calculated among host populations using the formula from Feder et al. (1993):

$$\text{AI}=\text{1 ‐}\;\left(\frac{\Sigma x_{i}y_{i}}{\sqrt{\Sigma x_{i}^{2}\cdot \Sigma y_{i}^{2}}}\right)\times 100$$

where *x*_i_ and *y*_i_ are the proportions of the total numbers of sexually active flies on day *i*. Newly eclosed cherry flies require 5–21 days to reach sexual maturity, depending upon temperature, with 7 days approximating conditions experienced by flies in nature (Frick et al., 1954). Adult flies can survive up to 30 days in the field, but can be reduced to 15 days if the average temperature exceeds 22°C (Frick et al., 1954). We thus considered adults to be sexually active either from 7 to 15 days or from 7 to 30 days posteclosion in our estimates of AI to encompass the probable upper and lower bounds of potential reproductive asynchrony between populations if they were to co‐occur nature.

### 2.3 Premating isolation

No‐choice mating experiments were performed to measure the degree of premating isolation between PNW, SW and ENA cherry flies (Table S2). Flies were collected in 2017 and 2018, with newly eclosing adults isolated by sex and population and maintained in cages with access to food (1:3 hydrolysed yeast protein and honey) and water for 7 days to reach sexual maturity. For each premating assay, six virgin male and six virgin female flies were placed in a clear quart‐sized container that contained a hanging 1‐inch‐diameter red cherry‐scented agarose sphere (1.0 g agarose: 2 g table sugar: 30 ml H_2_O: 10 ml cherry juice from concentrate) (Davis et al., 2011; Rull et al., 2010). Flies treated the spheres like fruit with adults mating on, and females ovipositing into the spheres. Copulating pairs lasting >5 min were removed from the mating cages and placed in their own separate cage for postmating isolation trials. New flies were added to the mating cages to maintain a constant number of six flies of each sex. Premating assays were performed over several days during daylight hours and observed for a minimum of 21.5 h per assay (Table S2). Flies were removed from mating cages and separated by sex at the end of each day before being reused the next day. Copulations were standardized by calculating the number of matings per hour for each assay. Premating RI was quantified using the formula RI_4A_ from Sobel and Chen (2014) designated here as:

$$\text{SI}=1‐2\times \left(\frac{H}{H+C}\right)$$

where *H* is the number or frequency of hybrid matings and *C* is the number of parental matings, such that *H*/(*H* + *C*) is the probability of gene flow *p*(GH) on a scale where 1 represents complete isolation, 0 represents random mating and −1 represents complete assortative mating.

### 2.4 Postmating isolation

To assess postmating isolation, we compared the mean numbers of eggs laid by females and the proportions of these eggs that hatched in pairwise crosses (hybrid and parental) between PNW, SW and ENA populations. Reduced fecundity may reflect poor sperm transfer and/or incompatibilities in the reproductive tracts of flies, and lower hatch rates may reflect intrinsic genetic incompatibilities affecting zygote development (Coyne & Orr, 2004). Postmating RI may exist in other life stages following egg hatch, but because of limitations in cherry fly husbandry, they could not be assessed at this time. Thus, our values for postmating RI are conservative, probably underestimating isolation among natural populations.

One to three mating pairs of flies were assembled in cages in 2018 and 2019 to measure postmating isolation. We established 1♀ × 1♂ cages using the successful copulating pairs from the premating isolation trials. For the 2♀ × 2♂ and 3♀ × 3♂ cages, we selected sexually mature, virgin females and males at random for the populations being evaluated. All cages in the postmating experiment contained food, water, a plastic leaf and a hanging agarose sphere. Agarose spheres were collected and replaced every 3 days. Eggs were removed from collected spheres, counted and placed on an agarose matrix in a 60 × 15‐mm Petri‐dish (1.0 g agarose: 2 g table sugar: 40 ml H_2_O) and monitored for egg hatch for 10 days. Each individual cross continued until >100 eggs were collected, or 4 weeks had elapsed, whichever occurred first. Parents from the crosses were frozen and stored at −80°C for genetic analysis.

To assess fecundity, we calculated the mean number of eggs laid per female per day in 1♀ × 1♂ crosses, totalling 194 crosses. To assess hatch rate, we calculated the proportion of eggs that hatched in all crosses that produced ≥10 eggs, totalling 217 crosses. Unmated *Rhagoletis* females can lay a small number of unfertilized eggs and, thus, setting a threshold of 10 eggs controlled for this (Opp & Prokopy, 1986). Significant differences in fecundity and egg hatch among pairwise cross permutations were tested for with nonparametric ANOVAs (Kruskal–Wallis tests) using eggs laid per female per day or proportion hatched as the response variable and crossed populations as the predictor. If significance was found, nonparametric *post hoc* Dunn's tests were performed, with a Benjamini–Yekutieli multicomparison *p*‐value adjustment.

### 2.5 Population‐level *Wolbachia* genotyping

*Wolbachia* strain diversity was assessed for sites: 1, 3–8 and 10–24 (Figure 1; Table S1) by genotyping 5–8 individuals from each site using target‐enrichment endosymbiont sequencing (TEEseq) (Schuler et al., 2019). TEEseq is a high‐throughput method that uses double digest restriction site‐associated DNA sequencing (ddRADseq) (DaCosta & Sorenson, 2014; Peterson et al., 2012) to target six genes used for multilocus sequence typing (MLST) of *Wolbachia* strains (Baldo et al., 2006), including the surface protein (*wsp*) and five other conserved genes (*gatB*, *coxA*, *hcpA*, *fbpA* and *ftsZ*). DNA was extracted from adult flies using QIAamp DNA Micro Kits (Qiagen). *Wolbachia* MLST sequences were PCR (polymerase chain reaction)‐amplified and Illumina libraries were prepared following Schuler et al. (2019). Sequence data were quality filtered, trimmed, and denoised using dada2 (Callahan et al., 2016) implemented in qiime2 (Bolyen et al., 2019). Reads were demultiplexed with cutadapt (Martin, 2011) and were sorted into unique amplicon sequencing variants (ASVs) in R using the packages *dada2* and *seqRFLP* (Ding & Zhang, 2012). Alignments of unique ASVs were individually checked and, when necessary, corrected manually, in codoncode aligner (Codon Code Corp.), with singletons removed from the final data set. Sequence data were deposited in the NCBI SRA (PRJNA747847) and Dryad (https://doi.org/10.5061/dryad.np5hqbztc).

### 2.6 Strain verification

Sanger sequencing was used to verify that the *Wolbachia* strains in the mating trials were identical to those identified with TEEseq genotyping. A 550‐bp fragment of *wsp* and a 500‐bp fragment of *hcpA* were PCR‐amplified (see Table S3 for primers and protocols) from 10 frozen parents from each population in the postmating RI assays. Base‐calling software does not disentangle two co‐occurring strains well (Tenney et al., 2007). Thus, we manually searched raw trace data using ugene (Okonechnikov et al., 2012) for the presence of strain‐specific polymorphisms. All sequenced flies were additionally genotyped for a 650‐bp fragment of the cytochrome *c* oxidase I (*COI*) mtDNA gene (Table S3). Reads were quality trimmed, aligned using muscle (Edgar, 2004), and manually checked using aliview (Larsson, 2014). We added two *R*. *cingulata* sequences (GenBank: HQ677090.1 and HQ677087.1) for comparison, as well as a *Rhagoletis completa* (Cresson) sequence as an outgroup (GenBank: HQ677111.1) (Doellman et al., 2019). We generated an RAxML (Stamatakis, 2014) tree for the *COI* alignment using a GTRGAMMA model. ggtree (Yu et al., 2017) was used to prepare trees. Fly *COI* sequences were deposited in GenBank (MZ820172–MZ820235).

### 2.7 Coupling of CI with nonendosymbiont induced RI

To examine the potential coupling of endosymbiont‐induced CI with nonendosymbiont‐related RI, we calculated the probability of gene flow *p*(GH) for a hypothetical scenario of secondary contact (currently all populations geographically isolated) using the data derived from our experiments on allochronic isolation and premating isolation, and those for Mexican populations from Tadeo et al. (2015). We then compared these estimates of gene flow to determine if they were greater or less than the critical migration rate (*m*_k_), defined as the migration rate above which a *Wolbachia* strain will introgress between hybridizing populations. Thus, if *p*(GH) > *m*_k_ the *Wolbachia* strain is expected to introgress and if *p*(GH) < *m*_k_ the *Wolbachia* strain is expected to remain diverged between populations. As derived by Flor et al. (2007), the critical migration rate for unidirectional CI with symmetrical migration is:

$$m_{k}=\;\text{min}\left\{\frac{c‐4t\left(1‐t\right)}{4t^{2}},\frac{4t\left(1+t‐2\sqrt{t}\right)}{{\left[\sqrt{c}+\sqrt{c‐4t\left(1‐t\right)}\right]}^{2}}\right\}$$

where *c* = the relative reduction in egg hatch for hybrid vs. parental crosses caused by CI (1 = no egg hatch for hybrids, 0 = equal egg hatch for hybrids), and *t* = the transmission rate of *Wolbachia* from mother to offspring (1 = completely efficient transfer, 0 = no transfer). For the case of bidirectional CI, the lower bound estimate for the critical migration rate derived by Telschow et al. (2005) is equal to:

$$m_{k}=\frac{2\left(a+b\right)‐ab‐\left(2\sqrt{{\left(a+b\right)}^{2}‐\left(a^{2}b\right)‐\left(b^{2}a\right)}\right)}{a^{2}}$$

where *a* = the level of CI caused by the strain having the largest effect on reducing egg hatch, and *b* = the level of CI caused by the strain having the lesser effect.

There are some important caveats in comparing *p*(GH) and *m*_k_. Most importantly, *m*_k_ considers migration with respect to two geographically distinct populations separated by an external, physical barrier restricting the proportion of immigrants contributing to *m*, often assuming a mainland–island model. In comparison, our estimates of *p*(GH) consider the effects of inherent, nonendosymbiont pre‐ and postmating RI on reducing gene flow relative to a baseline *m* of 0.5, reflecting panmixia in sympatry. Thus, gene flow may be lower than our estimates of *p*(GH) suggest, if following secondary contact, cherry‐infesting fly populations were to not fully geographically overlap. Consequently, the parameter space for maintaining *Wolbachia*‐induced CI may be more permissive than our calculations imply, and our estimates of *p*(GH) should be taken as a qualitative gauge for whether CI may be retained following contact. It is also possible, however, that host‐related forms of RI may weaken in contact zones due to hybridization and introgression compared to estimates derived between allopatric populations not experiencing gene flow. In this case, the potential for *Wolbachia* to spread between host populations may be greater than our than our estimates of *p*(GH) suggest.

## 3 RESULTS

### 3.1 Allochronic isolation

**FIGURE 2:**
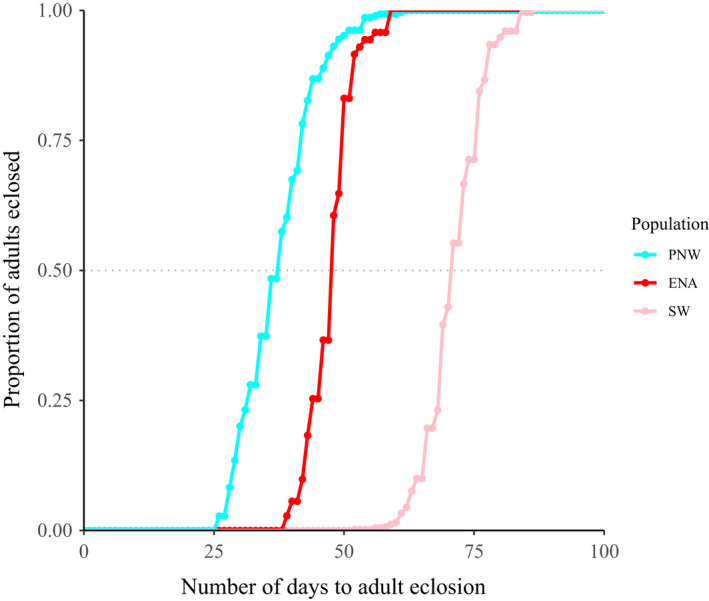
Cumulative adult eclosion curves for cherry flies collected in 2017 from the PNW (*n* = 289), ENA (*n* = 71) and SW (*n* = 579) populations. Days to eclosion is measured as the time it takes for adults to eclose once pupae are removed from their overwinter treatment. The dotted line denotes the median time of eclosion for PNW, ENA and SW flies

Cherry‐infesting fly populations differed significantly in their mean adult eclosion times (*F*_2,936_ = 3315.867, *R* ^2^ = 0.876, *p* < .001; Table S4), with *post hoc* tests indicating significant differences among all pairwise population comparisons (ENA × PNW: *p* < .001; ENA × SW: *p* < .001; PNW × SW: *p* < .001; Table S5). On average, *R. indifferens* adults from the PNW eclosed the earliest (30.68 ± 0.41 days [±SE], *n* = 289), followed by ENA flies (40.82 ± 0.51 days, *n* = 71), and then by SW flies (64.55 ± 0.23 days, *n* = 579) (Figure 2; see Figure S1A for eclosion curves including flies from the SMO [95.79 ± 1.93 days) and EVTM [55.67 ± 0.93 days]) based on data from (Tadeo et al., 2015). Assuming adults are sexually active 7–30 days posteclosion and if the differences in adult eclosion were maintained in sympatry, then levels of allochronic isolation (AI) would be: 28.5% for ENA vs. PNW, 86.5% for ENA vs. SW and 96.5% for PNW vs. SW (Table S6; Figure S1B). With a 15‐day adult lifespan, these estimates of *AI* would increase to 55.8%, 98.9% and 99.6%, respectively (Table S6; Figure S1C).

### 3.2 Premating isolation

No‐choice mating experiments revealed extensive premating isolation among PNW, SW and ENA fly populations. The highest level of premating isolation was observed between the PNW and ENA populations (SI =0.52), which are at the longitudinal ends of the cherry fly distribution. Slightly lower levels of premating isolation were detected between the SW and PNW populations (SI =0.41) or between the SW and ENA populations (SI =0.39). Premating isolation thus exists across the range of cherry flies in North America and there is a tendency for the degree of isolation to be related to the geographical distance separating populations.

### 3.3 Postmating isolation: fecundity

**FIGURE 3:**
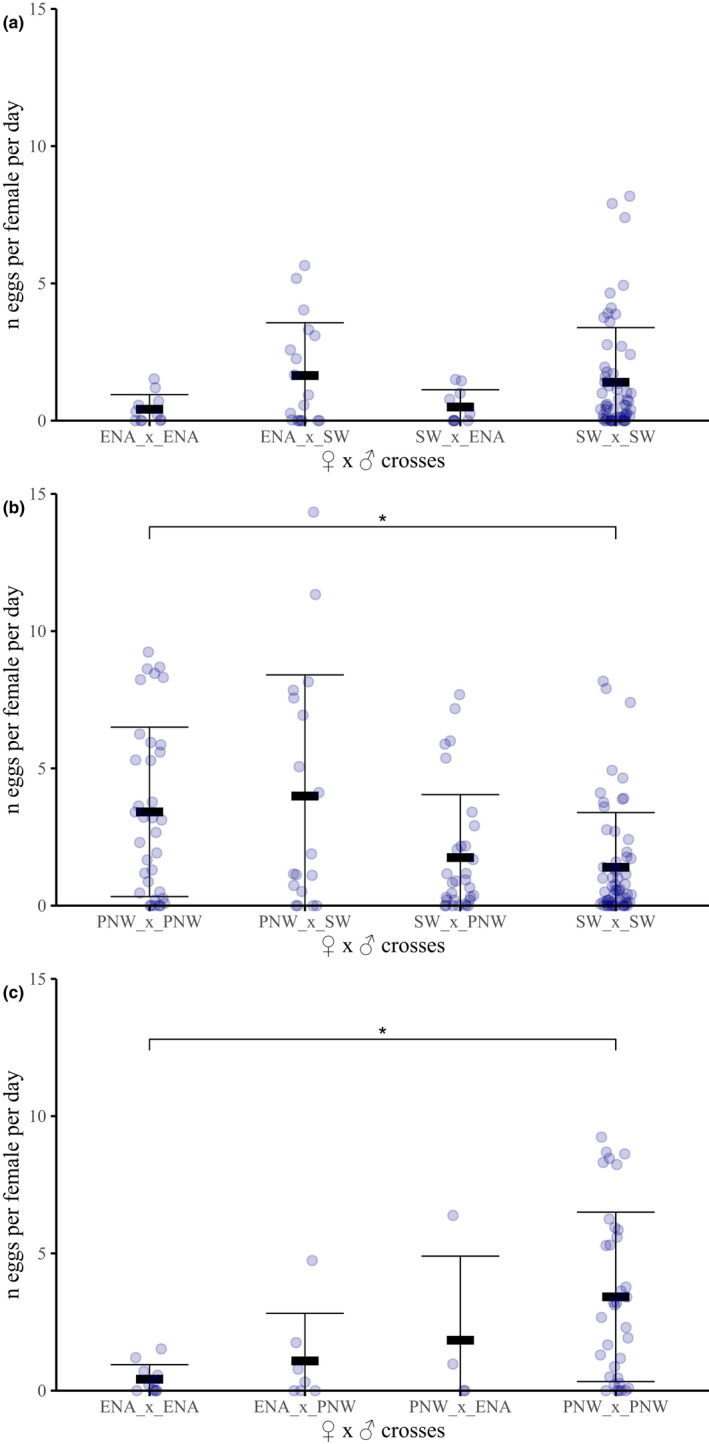
Fecundity (number of eggs laid per female per day) for pairwise cross combinations of females × males between: (a) SW × ENA; (b) PNW × SW; and (c) ENA × PNW flies. Stars denote significant differences in nonparametric pairwise tests between the two bracketed cross types (*adjusted *p* < .05). All other pairwise comparisons were not significant. Bars around means for cross types indicate one standard deviation (see Table S7 for means, standard errors, and sample sizes for each cross type and Tables S9 and S10 for *p*‐values)

Pairwise 1♀ × 1♂ parental and hybrid crosses between PNW, SW and ENA populations were assembled for fecundity analysis with between four and 60 replicates per treatment (Figure 3; Table S7). Nonparametric ANOVAs found no significant differences in fecundity between ENA × SW pairwise crosses (Kruskal–Wallis, *H*_3_ = 4.53, *p* = .21 Figure 3a; Table S8) but significant differences in fecundity among PNW × SW pairwise crosses (Kruskal–Wallis, *H*_3_ = 12.2, *p* = .007; Figure 3b; Table S8) and among PNW × ENA pairwise crosses (Kruskal–Wallis, *H*_3_ = 11.6, *p* = .009; Figure 3c; Table S8). Pairwise tests showed these differences in egg laying were primarily due to PNW females either being more fecund and/or having a greater propensity to oviposit into agarose spheres compared to SW (Table S9) and ENA females (Table S10), and not whether crosses were hybrid or parental.

### 3.4 Postmating isolation: egg hatch

**FIGURE 4:**
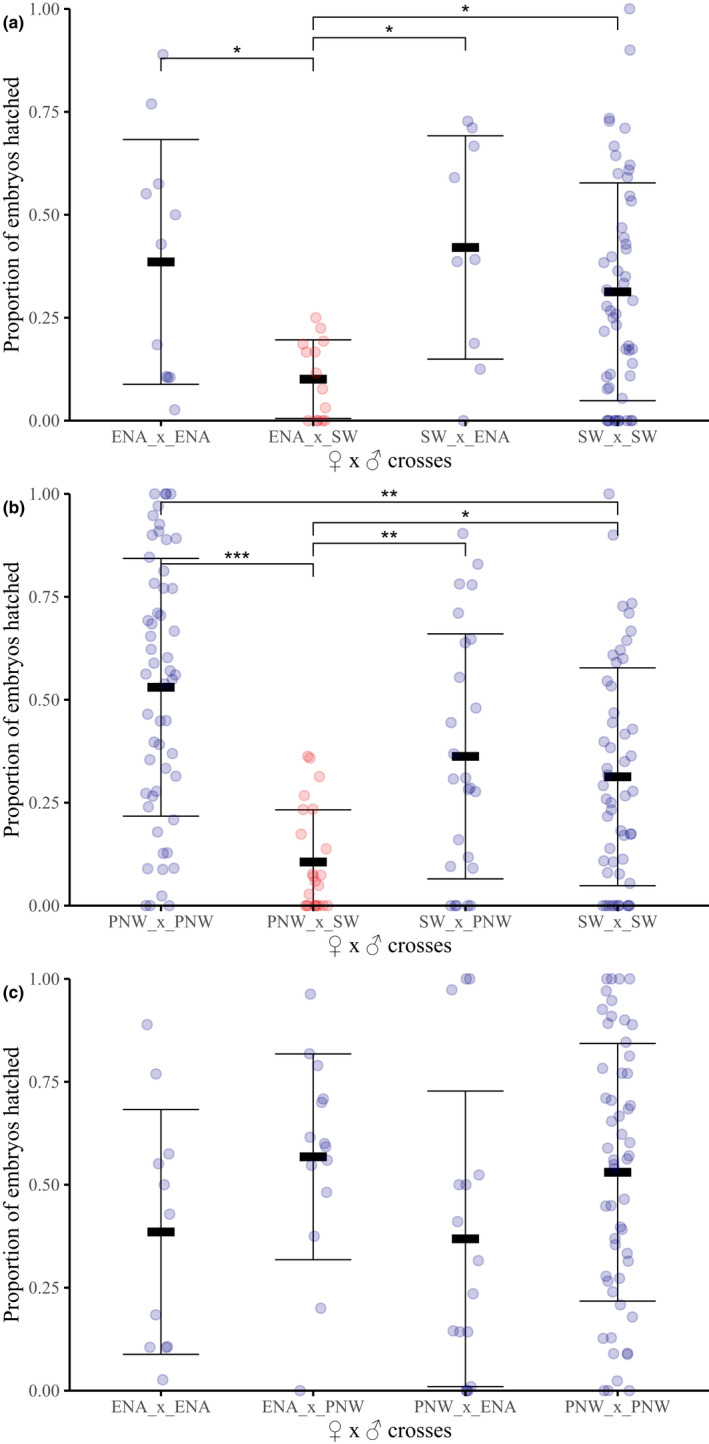
Proportions of eggs that hatched for different pairwise cross combinations of females × males between: (a) SW × ENA; (b) PNW × SW; and (c) ENA × PNW flies. Stars denote significant differences in nonparametric pairwise tests between the two bracketed cross types (*adjusted *p* <.05; **adjusted *p* <.01; ***adjusted *p* <.001). All other pairwise comparisons were not significant. Bars around means for cross types indicate one standard deviation (see Table S11 for means, standard errors and sample sizes for each cross type, and Tables S13 and S14 for *p*‐values)

Pairwise 1♀ × 1♂, 2♀ × 2♂ and 3♀ × 3♂ parental and hybrid crosses between PNW, SW and ENA flies were conducted for analysis of egg hatch with between nine and 54 replicates per treatment (Figure 4; Table S11). Nonparametric ANOVA showed significant differences in hatch rates among parental and hybrid cross types for matings between ENA × SW (Kruskal–Wallis, *H*_3_ = 11.59, *p* = .011; Figure 4a; Table S12), and PNW × SW flies (Kruskal–Wallis, *H*_3_ = 34.06, *p* < .001; Figure 4b; Table S12). In both cases, the significant reductions involved SW males. Egg hatch was 74% and 80% lower in hybrid crosses with male flies from the SW to females from the ENA and PNW flies, respectively, compared to parental crosses (Figure 4a,b; Tables S13 and S14). The reduction in egg hatch was unidirectional, as reciprocal hybrid crosses involving SW females with PNW or ENA males showed no significant difference in egg hatch compared to parental matings (Tables S13 and S14). No evidence for postmating isolation related to egg hatch was observed in either direction in PNW × ENA crosses (Kruskal–Wallis, *H*_3_ = 5.55, *p* = .136; Figure 4c; Table S12). Thus, the unidirectional pattern of postmating isolation related to egg hatch was associated with males possessing the SW/Mexican mtDNA haplotype and females the alternative PNW/ENA haplotype consistent with endosymbiont‐induced CI (LePage et al., 2017).

### 3.5 *Wolbachia* strain diversity

**FIGURE 5:**
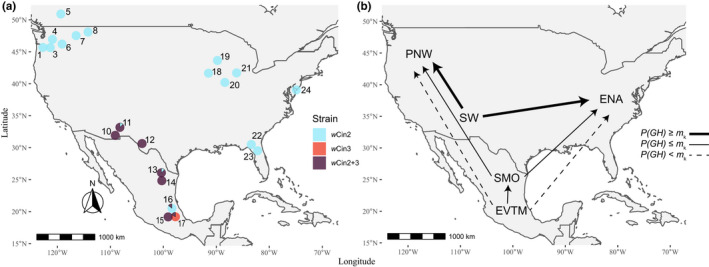
(a) Geographical distribution of *Wolbachia* strains *w*Cin2 and *w*Cin3 for (*n* = 152) cherry‐infesting *Rhagoletis* flies collected from 22 populations across North America. Populations 2 and 9 were not genotyped and were not included in this figure. Pie charts show the proportion of singly (*w*Cin2 or *w*Cin3) and doubly (*w*Cin2/*w*Cin3) infected individuals in populations based on TEEseq genotyping. Locality information for sites is given in Table S1 and data on TEEseq for cherry fly populations are provided in Table S15. (b) Estimated potential for introgression in the event of secondary contact for *Wolbachia* strain *w*Cin3 from the SW/Mexican populations to the ENA/PNW populations based on gene flow calculations and critical migration rates (*m*_k_) shown in Table S18. If *p*(GH) > *m*_k_ the *Wolbachia* strain is expected to introgress and if *p*(GH) < *m*_k_ the *Wolbachia* strain is expected to remain diverged between populations. Note that the probability of introgression varies depending on host lifespan and vertical transmission rate of the *Wolbachia* strain, *w*Cin3. Data for the Mexican populations were taken from Tadeo et al. (2015)

TEEseq generated 1,425,597 quality filtered reads (9503 reads per individual) for all five MLST barcoding genes and *wsp*. *Wolbachia* genotyping of 152 cherry‐infesting flies from 22 populations identified two major strains of *Wolbachia* (*w*Cin2 and *w*Cin3) across North America (Figure 5a; Table S15). Strain *w*Cin2, detected in all cherry fly populations, was previously described in *R*. *cingulata* populations in the SW and ENA, as well as in a recently introduced population in Europe (Schuler et al., 2013). Nucleotide blast results for the *wsp* gene showed that *w*Cin2 belongs to the *Wolbachia* supergroup A and is closely related to other *w*Mel‐like *Wolbachia* strains (Wolfe et al., 2021). The *w*Cin2 strain in the PNW and ENA were 100% identical based on the six MLST loci. A glycine to glutamic acid nonsynonymous substitution (GGA → GAA) at position 114 in the *hcpA* gene distinguished the *w*Cin2 strain in SW and Mexico populations from the PNW and ENA populations.

In contrast to *w*Cin2, strain *w*Cin3 was detected only in cherry‐infesting fly populations in the SW and Mexico (Figure 5a; Table S15), where it normally co‐infected flies along with *w*Cin2. Strain *w*Cin3 coincided with a unique mtDNA haplotype and the observed unidirectional reduction in egg hatch in hybrid crosses involving SW males, implying that this strain is probably inducing unidirectional CI. A nucleotide blast search based on the *wsp* gene showed that *w*Cin3 belongs to the *Wolbachia* B supergroup. Sanger sequencing verified that PNW and ENA flies used in the crossing experiments above harboured only strain *w*Cin2, while SW flies possessed both *w*Cin2 and *w*Cin3 (Table S16). *COI* barcoding also confirmed that SW flies had a different mitochondrial haplotype from the PNW and ENA flies, following the previously identified pattern from Doellman et al. (2019) (Figure S2; Table S16).

All cherry‐infesting flies genotyped in the current study were infected by at least one strain of *Wolbachia*. In individuals co‐infected with *w*Cin2 and *w*Cin3, strain *w*Cin3 accounted for a smaller mean portion of TEEseq reads than *w*Cin2, which varied from 3.2% to 31.2% depending on population (Table S17), suggesting that *w*Cin3 is present at a lower titre than *w*Cin2. It is possible that differences in the titre of *w*Cin3 could contribute to the incomplete CI we observed in our crossing assays, a hypothesis requiring further testing. Strain *w*Cin2, previously thought to be fixed in all North American cherry flies (Schuler et al., 2013), was not present in five of six individuals (83.3%) screened from site 17 in the EVTM (Figure 5a; Table S15). All six individuals (100%) at site 17 possessed the *w*Cin3 strain. In contrast, *w*Cin3 was not present in all flies in the SW and Mexico, absent in one of eight flies (12.5%) from site 11 in the SW, one of eight flies (12.5%) from site 13 in the SMO, and six of seven flies (85.7%) from site 16 in the EVTM (Figure 5a; Table S15).

### 3.6 Coupling of endosymbiont‐ and nonendosymbiont‐related RI

Depending upon parameter values, current levels of pre‐ and postmating RI are probably insufficient to stop the spread of *w*Cin3 from the SW to the rest of the USA following a hypothetical secondary contact scenario (Figure 5b; Table S18). Based on the reduction of egg hatch for hybrid vs. parental crosses, *Wolbachia*‐induced CI in matings between SW males (co‐infected with *w*Cin3) and PNW females and ENA females (both lacking *w*Cin3) was equal to 0.800 and 0.738, respectively (Table S18). Given these values of CI, lower bound estimates of *m*_k_ to impede the introgression of *w*Cin3 based on unidirectional incompatibility would range from ~3 × 10^−5^ (for a transmission rate *t* of 0.99) to ~7 × 10^−3^ (for a transmission rate *t* of 0.875). We currently do not know the value of *t* for cherry‐infesting flies, but vertical transmission is typically high in insects (with rare exceptions), falling in the range of 0.99 to 0.90 (Carrington et al., 2011; Hague et al., 2020; Hoffmann et al., 2014; Kriesner et al., 2016). We included 0.875 as a lower vertical transmission estimate based on our TEEseq data, showing one of eight flies from the SW population at site 11 and site 13 lost *w*Cin3 in our genotype survey.

In comparison, *p*(GH) from nonendosymbiont‐related RI between SW and PNW populations, assuming a mean adult longevity of 15 days, is estimated at 6.342 × 10^−4^ (= 0.00215 [due to allochronic isolation] × 0.295 [due to sexual isolation]) and 1.612 × 10^−3^ between the SW and ENA (= 0.00523 × 0.307; Table S18). Under a 30‐day adult lifespan, *p*(GH) increased to 5.163 × 10^−3^ and 2.066 × 10^−2^, respectively. Thus, estimates of *p*(GH) approximate but are not definitively below the minimal estimates of *m*_k_ required to curtail the potential spread of *w*Cin3 from the SW. Consequently, if vertical transmission is efficient, adult lifespans are long, and nonendosymbiont RI were to weaken in hybrid zones, then *w*Cin3 would probably introgress from the SW into the PNW and ENA following secondary contact.

## 4 DISCUSSION

Our goal in the current study was to determine whether endosymbiont‐induced CI exists in cherry flies and whether it could potentially couple with other forms of host‐related RI to contribute to speciation. To answer these questions, we focused on four aims. For aim 1, we demonstrated that varying degrees of pre‐ and postmating RI presently exist among cherry‐infesting fly populations across North America. For aim 2, we found a pattern of unidirectional postzygotic isolation in hybrid crosses between SW males and females from the ENA and PNW. For aim 3, our genomic survey indicated that a unique strain of *Wolbachia*, *w*Cin3, occurs in SW and Mexican populations, corresponding to the observed postzygotic isolation and disjunct mtDNA haplotype found in SW and Mexican populations. This is a pattern consistent with unidirectional *Wolbachia*‐induced CI. Finally, for aim 4, we calculated that the coupling of nonendosymbiont RI with endosymbiont CI should not prevent gene flow and the spread of *w*Cin3 in the event of secondary contact between SW and ENA or PNW fly populations.

### 4.1 Evidence for *Wolbachia*‐induced CI

Our findings provide strong support for endosymbiont‐induced CI in cherry‐infesting fly populations and implicate a *Wolbachia* B supergroup strain, *w*Cin3, as its causative agent. Attempts at rearing larvae from host fruit treated with antibiotics to cure *Wolbachia* and to rescue the CI phenotype in cherry flies have been unsuccessful to date, hindering a direct verification that *w*Cin3 is responsible for the unidirectional reduction in egg hatch. It might be possible that the observed CI phenotype is due to a different endosymbiont whose presence/absence corresponds to that of *w*Cin3 (Perlman et al., 2014) or perhaps is due to mitochondrial–nuclear incompatibilities (Telschow et al., 2019). Our finding that various individuals in the SW and Mexico lack *w*Cin3 but possess the derived SW and Mexican mtDNA haplotype makes it possible to perform crosses in the future to strengthen empirical support for CI induced by *w*Cin3. We further note that our genetic survey of different *Wolbachia* strains in the USA and Mexico also corresponds to patterns of postzygotic isolation reported by Tadeo et al. (2015) in crosses among SMO, EVTM and ENA flies, lending further empirical support to *Wolbachia* strain *w*Cin3 as the causative agent of CI.

### 4.2 Coupling of CI with other RI barriers

Our results are equivocal as to whether *Wolbachia*‐induced CI is currently coupled strongly enough with other identified forms of host‐related pre‐ and postmating RI to stop the spread of *w*Cin3 across North America if SW flies were to re‐establish contact with flies from the PNW and ENA. If vertical transmission is reduced and adult longevity is limited, then endosymbiont differences may remain. However, if not, then *w*Cin3 would probably spread. An argument could still be made that if cherry‐infesting flies in the USA were to continue to remain allopatric or were to exchange migrants at a low rate following contact, then *Wolbachia* could be considered to contribute to ongoing RI and speciation. However, it is also possible that nonendosymbiont‐related RI may weaken following contact. For example, allochronic isolation could decrease if regional differences in the timing of fly eclosion were to diminish due to the fruiting times of host cherries converging in contact areas, facilitating the introgression of *w*Cin3. In this regard, a reduced coupling of RI barriers in hybrid zones may restrict *Wolbachia's* role in speciation to systems in which divergence occurs primarily or exclusively in allopatry.

Cytoplasmic‐ and nuclear‐related RI is greater, however, between Mexican and both ENA and PNW populations than between SW and both ENA and PNW populations (Figure 5b; Table S18). Thus, if flies from Mexico were to come into secondary contact with those from the USA, a stronger case could be made that CI would persist and contribute to population divergence. Tadeo et al. (2015) measured eclosion times, fecundity and egg hatch rates from hybrid matings among populations from the SMO (site 14), EVTM (site 17) and ENA (Kearneysville, West Virginia). SMO and EVTM flies eclose later than flies from populations in the USA (Tadeo et al., 2015), generating higher levels of *AI* (Figure S1; Table S18). A reduced hatch rate was observed unidirectionally in crosses between SMO males and ENA females (Tadeo et al., 2015). As a result, estimates of *p*(GH) are equivalent to or below the *m*_k_ value needed to retain the *Wolbachia w*Cin3 difference between fly populations in the SMO and ENA/PNW (Figure 5b; Table S18). A reduced hatch rate was also observed bidirectionally in crosses between EVTM and ENA flies and unidirectionally between EVTM females and SMO males (Tadeo et al., 2015). These results correspond to our observation that the majority of individuals from the EVTM population used by Tadeo et al. (2015) (site 17) may not have been infected with *w*Cin2 and were singly infected by *w*Cin3 (Figure 5a; Table S15). Consequently, our estimates of *p*(GH) between EVTM and ENA/PNW populations fall below the *m*_k_ value derived from the bidirectional incompatibility formula of Telschow et al. (2007) needed to maintain *Wolbachia* strain differences between the EVTM and ENA populations (Figure 5b; Table S18). Estimates of *p*(GH) are also equivalent to or below the unidirectional *m*_k_ value between the EVTM and the SMO populations (Figure 5b; Table S18).

### 4.3 *Wolbachia* hybrid zones

Although the majority of *Wolbachia* infections do not appear to generate CI (Hoffmann et al., 2015), enough examples exist that the endosymbiont could potentially contribute to RI between host species. Our results thus add to an increasing list of studies implicating *Wolbachia* as a causative agent of CI between diverging taxa (Bordenstein et al., 2001; Cooper et al., 2017; Gebiola et al., 2016; Miller et al., 2010; Shoemaker et al., 1999). The question then is whether *Wolbachia* or any other endosymbiont‐induced CI can become coupled with other forms of RI as populations transition from fully interbreeding demes to partially isolated taxa to fully diverged species. It is important to note that all cherry‐infesting fly populations in our study are allopatric, where none of the populations possessing different *Wolbachia* strains geographically overlap. Thus, despite seeming to represent different stages from weak to strong coupling between cytoplasmic and nuclear‐based RI, we have no empirical evidence to verify that CI would be maintained if populations were to hybridize following secondary contact.

Surprisingly, the same is also true for almost all other *Wolbachia* CI systems in insects. While examples exist demonstrating the spread of *Wolbachia* through populations (Bakovic et al., 2018; Kriesner et al., 2013; Raychoudhury et al., 2010; Riegler et al., 2005; Schuler et al., 2016) and its transmission between species (Cooper et al., 2019; Miyata et al., 2020; Morrow et al., 2014; Rousset & Solignac, 1995; Schuler et al., 2013; Turelli et al., 1992), there is little evidence for the stable maintenance of *Wolbachia*‐induced CI across a hybrid zone. One possible example involves two different subspecies of *Chorthippus parallelus* grasshoppers that came into secondary contact in the Pyrenees Mountains of Europe following the retreat of glaciers ~9000 years ago (Martínez‐Rodríguez & Bella, 2018). However, the story is biogeographically complex, as hybrid populations of viable and fertile hybrid grasshoppers exist in restricted areas, where they are sufficiently geographically separated such that secondary contact is unlikely (Martínez‐Rodríguez & Bella, 2018). In these grasshoppers, one supergroup B strain of *Wolbachia* appears to have been recently acquired and has spread from continental Europe into the Iberian Peninsula. In contrast, there are different supergroup F *Wolbachia* strains between the *C*. *parallelus* subspecies with regional/local variation among sites and with a putative recombinant strain present in the hybrid zone (Martínez‐Rodríguez & Bella, 2018). Thus, *C*. *parallelus* does not correspond to a simple geographically continuous hybrid zone, begging the question of why there appears to be a dearth of such examples.

One possible explanation accounting for the paucity of stable *Wolbachia* hybrid zones is that the coupling of inherent isolating barriers may weaken in these areas following secondary contact such that the endosymbiont will eventually introgress between populations. To prevent introgression, vertical transmission rates must also be incomplete or, in the case of bidirectional CI, not differ between strains, otherwise *Wolbachia* differences will be lost between hybridizing populations (Turelli, 2010; Turelli & Hoffmann, 1995). In addition, *Wolbachia* strains must not impart positive fitness benefits to their hosts, as this will increase their rate of spread (Fry et al., 2004). Finally, horizontal transfer by means other than hybridization must not occur frequently enough for *Wolbachia* differences between taxa to eventually dissipate in sympatry (Sanaei et al., 2021). Thus, even when species are strongly reproductively isolated, *Wolbachia* differences may not persist for prolonged periods of time when populations broadly overlap. If true, then stable *Wolbachia* hybrid zones may be rare in nature. Moreover, when observed, they may be recent in origin and composed of a patchwork of largely isolated subpopulations experiencing restricted migration, such as *C*. *parallelus*, rather than being contiguous and varying in a smooth clinal fashion. Consequently, estimates of *m*_k_, which assume a mainland–island model of migration (e.g. Flor et al., 2007; Telschow et al., 2002, 2005), may reflect elements of the spatial structure of hybrid zones needed to slow the spread of *Wolbachia* and allow for the continued participation of CI in speciation.

## 5 CONCLUSIONS

In the current study we ask whether *Wolbachia*‐induced CI contributes to speciation, with specific reference to the coupling of cytoplasmic and nuclear‐related forms of RI in cherry‐infesting *Rhagoletis* flies. Clearly, if cherry‐infesting fly populations were to remain in allopatry throughout the entirety of their divergence, then endosymbiont‐caused CI could be regarded as a factor contributing to the reproductive isolation of these species. The answer becomes more complicated though if cherry flies were to come into secondary contact and hybridize, which is of general importance as many taxa may experience gene flow at some stage of their divergence (Nosil, 2008). The issue then is whether and how CI can become coupled with other host‐related RI barriers across hybrid zones to maintain population differences. *Wolbachia* strains can be acquired horizontally (Sanaei et al., 2021) or eventually lost (Bailly‐Bechet et al., 2017) and, thus, in most cases, the long‐term prognosis for the retention of *Wolbachia*‐induced CI following secondary contact may be poor. This does not mean that if eventually lost, the endosymbiont did not play a role in speciation. For example, during the time it was present, *Wolbachia*‐induced CI could have maintained population divergence and facilitated the evolution of additional RI, for example, through the process of reinforcement (Jaenike et al., 2006). The difficulty may come in finding direct empirical evidence supporting the involvement of the endosymbiont during such stages, particularly if the structure of hybrid zones following secondary contact is more conducive to the spread rather than retention of *Wolbachia*. Our analysis of the coupling of nonendosymbiont RI with *Wolbachia* CI provides a useful empirical framework based on theory to help quantify the involvement of *Wolbachia* and other endosymbionts in the process of speciation.

## CONFLICT OF INTERTEST

All authors declare no conflict of interest.

## AUTHOR CONTRIBUTIONS

Sample collection: DJB, HS, MMG, JM, CT, WLY, JR, MA, GRH, RG and JLF. Fly husbandry: DJB, HS, MMG, JM, CT, WLY, JR, MA and RG. DNA extractions: DJB, HS and JM. Library preparation: HS and MMD. Bioinformatics; DJB, HS and MMD. Statistical analysis: DJB, HS, MMD and JLF. Writing: DJB, HS, TMW, PN and JLF. Study design: DJB, HS, JLF. Scientific contributions: TMW, WLY, MA, GRH, CS and PN. Project supervision: JLF.

## Supporting information

Fig S1‐S2

Table S1‐S18

## Data Availability

All data associated with this study have been uploaded to Dryad (https://doi.org/10.5061/dryad.np5hqbztc). Additionally, raw sequence data were uploaded to the NCBI SRA (PRJNA747847) and *COI* sequences were uploaded to GenBank (MZ820172–MZ820235).
