# Supplementary material for: Testing the potential contribution of Wolbachia to speciation when cytoplasmic incompatibility becomes associated with host‐related reproductive isolation
Source: Mol Ecol. 2021 Sep 16;31(10):2935–50. doi: 10.1111/mec.16157 (PMC9290789; doi:10.1111/mec.16157)
Supplement: Supplementary file 1 — Fig S1‐S2 [file MEC-31-2935-s002.docx]

##
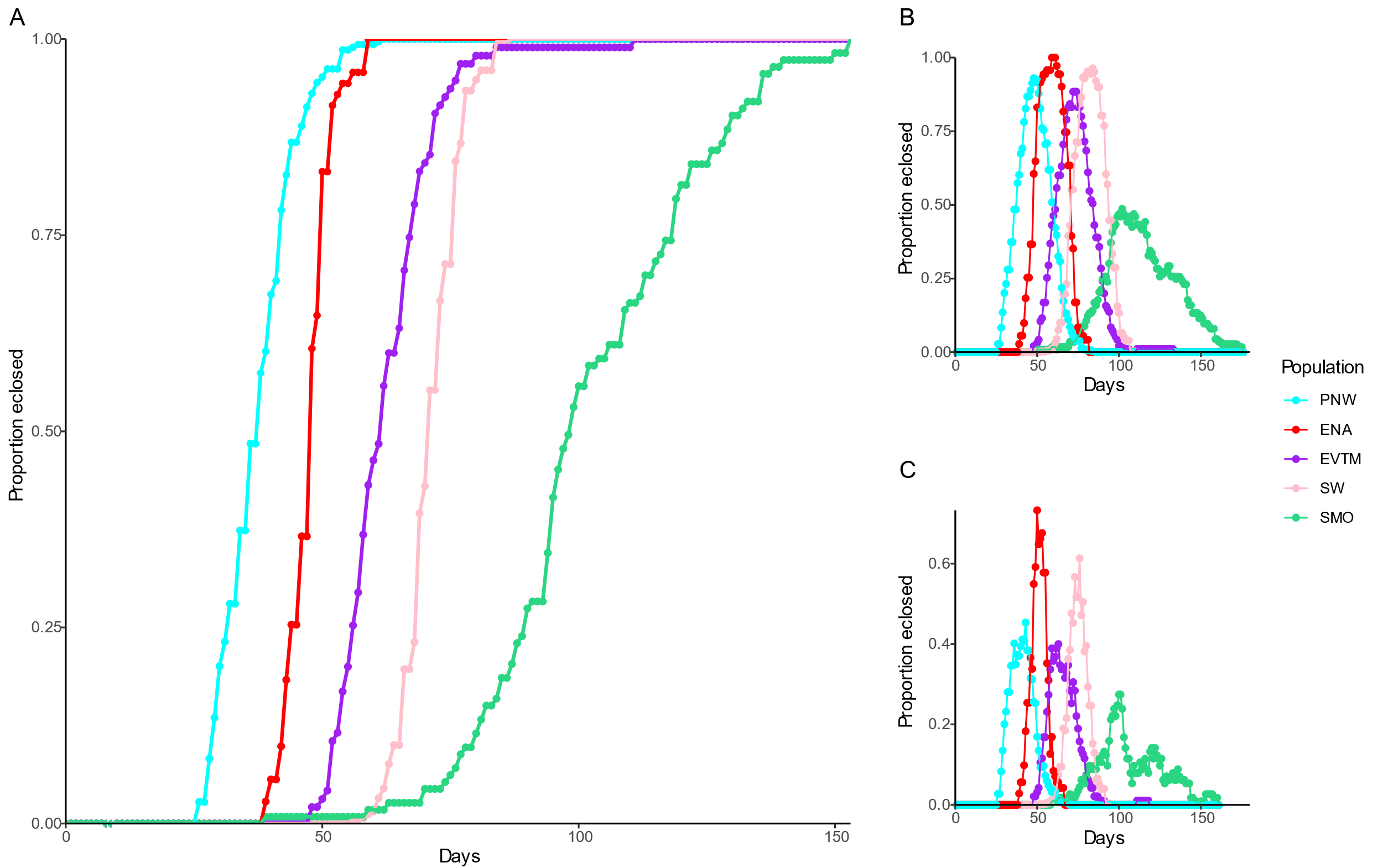


**Figure S1.** (A) Cumulative adult eclosion curves for PNW, ENA, EVTM, SW, and SMO cherry fly populations. Mean for PNW = 30.68 + 0.41 days s.e., n = 289; ENA = 40.82 *+* 0.51 days s.e., n = 71; EVTM = 55.67 *+* 0.93 days s.e., n = 95; SW = 64.55 *+* 0.23 days s.e., n = 579; and SMO = 95.79 *+* 1.93 days s.e., n = 113. (B) Active population overlap assuming seven days to sexual maturity and a 30-day lifespan. (C) Active population overlap assuming seven days to sexual maturity and a 15-day lifespan. Data for the two Mexican populations in the SMO and EVTM come from Tadeo et al. (2015).


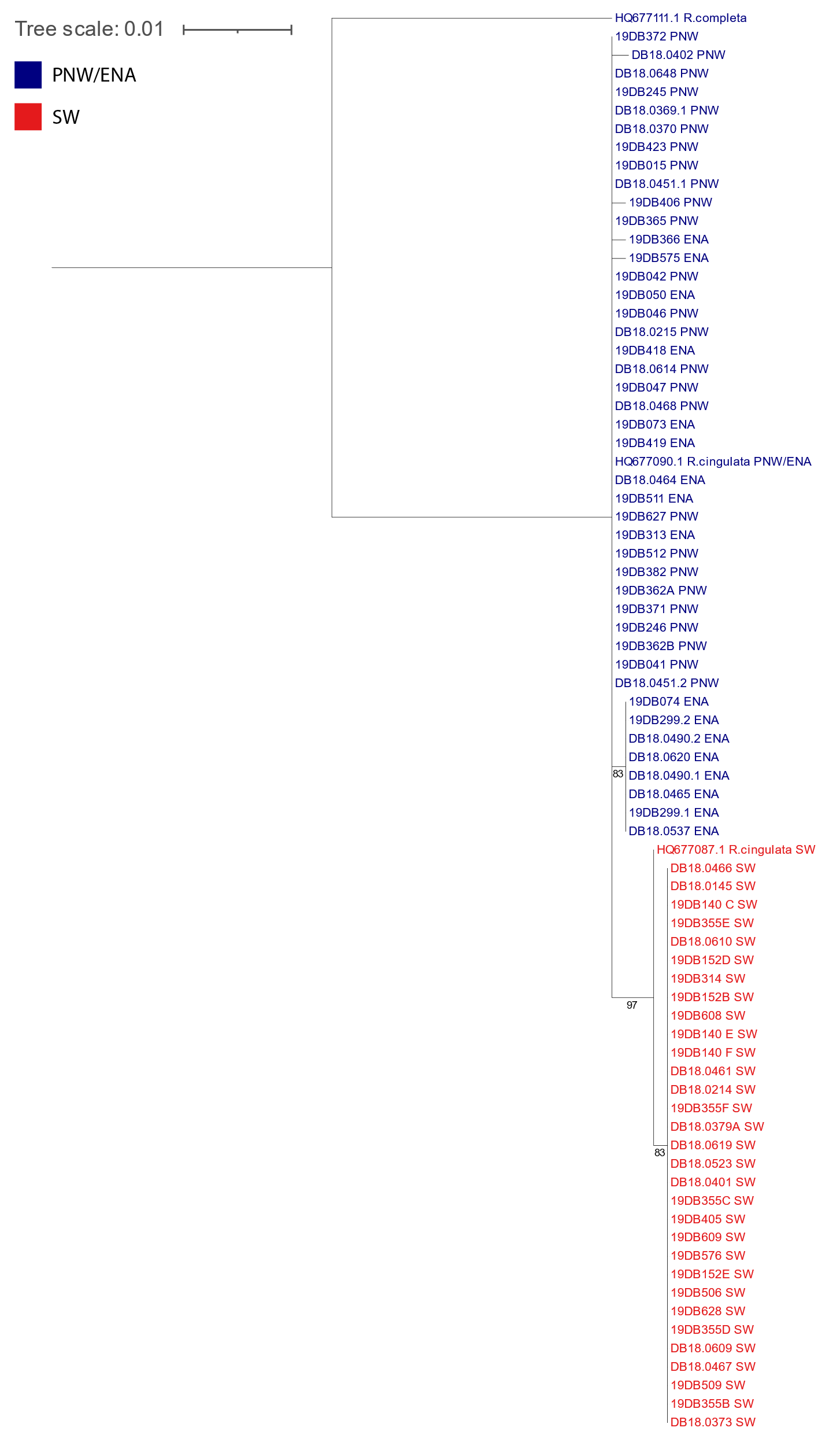


**Figure S2**. Rooted mtDNA tree constructed in RAxML for a 650bp fragment of the Cytochrome c oxidase I (*COI*) gene. Bootstrap support values are given on branches. The tree is rooted using a *R. completa* sequence (GenBank: HQ677111.1). Haplotypes in blue represent those sequenced from *R. indifferens* in the PNW and *R. cingulata* in the ENA, while those in red denote *R. cingulata* from the SW.

**References**

Tadeo, E., Feder, J. L., Egan, S. P., Schuler, H., Aluja, M., & Rull, J. (2015). Divergence and evolution of reproductive barriers among three allopatric populations of *Rhagoletis cingulata* across eastern North America and Mexico. *Entomologia Experimentalis et Applicata*, *156*(3), 301–311. doi: 10.1111/eea.12331
