# Supplementary material for: Testing the potential contribution of Wolbachia to speciation when cytoplasmic incompatibility becomes associated with host‐related reproductive isolation
Source: Mol Ecol. 2021 Sep 16;31(10):2935–50. doi: 10.1111/mec.16157 (PMC9290789; doi:10.1111/mec.16157)
Supplement: Supplementary file 2 — Table S1‐S18 [file MEC-31-2935-s001.docx]

## **Supplementary Tables**

**Table S1.** Collection sites analyzed in the study. Site numbers (Site #) correspond to those appearing in Fig. 1. Pop. refers to which of the five general regional populations that a site resides in: PNW = Pacific Northwest USA; SW = Southwestern USA; SMO = Sierra Madre Oriental Mountains Mexico; EVTM = Eje Volcánico Trans Mexicano and ENA = Eastern USA (ENA). Information regarding the species collected at sites, site locations, and site latitude and longitude, are also listed. Crossing experiments were done using flies from sites: 1, 2, 9, 10, 12, and 21.

| Site # | Pop. | Species | Locality | Latitude | Longitude |
| --- | --- | --- | --- | --- | --- |
| 1 | PNW | *R. indifferens* | Vancouver, WA | 45.68281 | -122.599 |
| 2 | PNW | *R. indifferens* | Hillcrest Rd, Hood River, OR | 45.58266 | -121.557 |
| 3 | PNW | *R. indifferens* | Aplin Rd., White Salmon, WA | 45.78332 | -121.505 |
| 4 | PNW | *R. indifferens* | Nile, WA | 46.8202 | -120.94 |
| 5 | PNW | *R. indifferens* | Salmon Arm, BC, Can | 50.915 | -119.357 |
| 6 | PNW | *R. indifferens* | Kennewick, WA | 46.20719 | -119.127 |
| 7 | PNW | *R. indifferens* | Rose Lake, ID | 47.53869 | -116.473 |
| 8 | PNW | *R. indifferens* | Somers, MT | 48.09 | -114.23 |
| 9 | SW | *R. cingulata* | Oak Creek, AZ | 34.787 | -111.76 |
| 10 | SW | *R. cingulata* | Chiricahua Mtns, AZ | 31.92889 | -109.088 |
| 11 | SW | *R. cingulata* | Gila Cliff Dwelling Mon., NM | 33.17889 | -108.204 |
| 12 | SW | *R. cingulata* | Davis Mountains, TX | 30.64639 | -104.019 |
| 13 | SMO | *R. cingulata* | Nuevo León, Mex | 25.81333 | -100.367 |
| 14 | SMO | *R. cingulata* | Los Lirios, Coahuila, Mex | 25.34944 | -100.296 |
| 15 | EVTM | *R. cingulata* | Tlalpan, Distrito Federal (CDMX), Mex | 19.132127 | -99.090141 |
| 16 | EVTM | *R. cingulata* | Tulancingo, Hidalgo, Mex | 20.1343 | -98.3802 |
| 17 | EVTM | *R. cingulata* | Huamantla, Tlaxcala, Mex | 19.315 | -97.8744 |
| 18 | ENA | *R. cingulata* | Scott Park, Iowa City, IO | 41.653 | -91.48 |
| 19 | ENA | *R. cingulata* | The Dells, Wisconsin Dells, WI | 43.643 | -89.783 |
| 20 | ENA | *R. cingulata* | Lake of the Woods, IL | 40.20003 | -88.3846 |
| 21 | ENA | *R. cingulata* | South Bend, IN | 41.698 | -86.172 |
| 22 | ENA | *R. cingulata* | Live Oak, Suwannee County, FL | 30.36833 | -83.2439 |
| 23 | ENA | *R. cingulata* | Gainesville, FL-Health, FL | 29.644 | -82.355 |
| 24 | ENA | *R. cingulata* | Green Creek, Cape May, NJ | 39.03639 | -74.9014 |

**Table S2.**  Observation time in hours (# Obs. hrs.) for pairwise crosses performed between flies from PNW, SW, and ENA populations. All assays were observed for a minimum of 21.5 hours.

| **Female population** | **Male population** | | **# Obs. hrs.** |
| --- | --- | --- | --- |
| ENA | | ENA | 21.5 |
| PNW | | PNW | 22.5 |
| ENA | | PNW | 22 |
| PNW | | ENA | 29 |
| SW | | SW | 72 |
| SW | | ENA | 50 |
| ENA | | SW | 20 |
| SW | | PNW | 35 |
| PNW | | SW | 28 |

**Table S3.** Forward (F) and Reverse (R) PCR primer pairs used in confirmatory Sanger DNA sequencing of cherry fly mtDNA and *Wolbachia* *wsp* and *hcpA* genes. PCR was performed in a 15 μl volume containing 1.5 μl 10X Taq buffer, 2.0 mM MgCl2, 100 μM dNTPs, 0.4 U Taq polymerase (Bulldog Bio; Portsmouth, NH), 0.3 μM forward primer, 0.3 μM reverse primer, and 1 μl DNA template. PCR conditions for mtDNA: 94°C for 1 min, 35 cycles of: 94°C for 30 s, 52°C for 50 s, and 72°C for 1 min, and a final extension period of 72°C for 10 min. The thermocycler profile for *wsp*: 94°C for 2 min, 35 cycles of: 94°C for 30 s, 55°C for 45 s, and 72°C for 1 min, and a final extension period of 72°C for 15 min. Finally, the thermocycler profile for *hcpA*: 94°C for 3 min, 30 cycles of: 94°C for 30 s, 50°C for 55 s, and 72°C for 1 min, and a final extension period of 72°C for 10 min.

| **Locus** | **Name** | **Dir.** | **Sequence** | **Length (bp)** | **Author** |
| --- | --- | --- | --- | --- | --- |
| *COI* | UEA5 | F | AGTTTTAGCAGGAGCAATTACTAT | 780 | Lunt et al. (1996) |
| *tRNA leu* | UEA10 | R | TCCAATGCACTAATCTGCCATATTA | 780 | Lunt et al. (1996) |
| *wsp* | *wsp*_F1 | F | GTCCAATARSTGATGARGAAAC | 590 | Baldo et al. (2006) |
| *wsp* | *wsp*_R1 | R | CYGCACCAAYAGYRCTRTAAA | 590 | Baldo et al. (2006) |
| *hcpA* | hcpA_F1 | F | GAAATARCAGTTGCTGCAAA | 500 | Baldo et al. (2006) |
| *hcpA* | hcpA_R1 | R | GAAAGTYRAGCAAGYTCTG | 500 | Baldo et al. (2006) |

**Table S4**. ANOVA testing for significant effects of population (PNW, SW, ENA) on mean adult eclosion time, with flies from the ENA representing the intercept.

|  | |
| --- | --- |
|  | *Dependent variable:* |
|  |  |
|  | Days to Eclosion |
|  | |
| Pop. PNW | -10.135^***^ |
| Pop. SW | 23.729^***^ |
|  |  |
|  |  |
| Intercept: Pop. ENA | 40.817^***^ |
|  |  |
|  |  |
|  | |
| Observations | 939 |
| R^2^ | 0.876 |
| Adjusted R^2^ | 0.876 |
| Residual Std. Error | 5.905 (df = 936) |
| F Statistic | 3,315.867^***^ (df = 2; 936) |
|  | |
|  | * p<=0.05; ** p<=0.01; *** p<=0.001 |

**Table S5**. Tukey pairwise comparisons testing for significant differences in adult eclosion times between cherry fly populations from different geographic regions (ENA vs PNW, ENA vs. SW, PNW vs. SW).

| **group1** | **group2** | **null** | **estimate** | **conf.low** | **conf.high** | **p.adj** | **p.adj.signif** |
| --- | --- | --- | --- | --- | --- | --- | --- |
| ENA | PNW | 0 | -10.13524051 | -11.9713459 | -8.299135117 | 2.90E-13 | **** |
| ENA | SW | 0 | 23.72886716 | 21.98580581 | 25.47192851 | 2.90E-13 | **** |
| PNW | SW | 0 | 33.86410767 | 32.86572712 | 34.86248821 | 2.90E-13 | **** |

**Table S6.** Estimated allochronic isolation (*AI* *=* % non-overlap in seasonal distributions of mating times of flies based on adult eclosion curves) between pairs of cherry fly populations from the PNW, SW, ENA, EVTM, and SMO, as derived from the formula of Feder, Hunt, and Bush (1993). Shown are results assuming a 7-day period to sexual maturation following eclosion and either a 15-day or 30-day mean adult longevity in nature. Eclosion data used to estimate *AI* for flies from the SMO and EVTM come from Tadeo et al. (2015).

| **Pop. A** | **Pop. B** | **15 Day** | **30 Day** |
| --- | --- | --- | --- |
| PNW | SW | 99.57 | 96.50 |
| PNW | ENA | 55.82 | 28.52 |
| PNW | EVTM | 92.65 | 77.59 |
| PNW | SMO | 99.89 | 98.70 |
| ENA | SW | 98.95 | 86.54 |
| ENA | EVTM | 71.57 | 43.45 |
| ENA | SMO | 99.67 | 97.13 |
| SW | EVTM | 41.14 | 22.68 |
| SW | SMO | 80.62 | 68.08 |
| EVTM | SMO | 89.89 | 82.57 |

**Table S7.** Mean + s.e. of number of eggs laid per female per day for pairwise cross combinations between PNW, SW, and ENA populations. 194 1♀ × 1♂ crosses from 2018 and 2019 were used to assess fecundity, where n represents the number of different crosses in a given cross type.

| **♀ pop.** | **♂ pop.** | **Mean** | **s.e.** | **n** |
| --- | --- | --- | --- | --- |
| ENA | ENA | 0.42 | 0.16 | 11 |
| ENA | SW | 1.64 | 0.45 | 18 |
| SW | ENA | 0.50 | 0.20 | 10 |
| SW | SW | 1.40 | 0.26 | 60 |
| PNW | PNW | 3.42 | 0.52 | 35 |
| PNW | SW | 3.99 | 1.04 | 18 |
| SW | PNW | 1.75 | 0.41 | 31 |
| ENA | PNW | 1.08 | 0.66 | 7 |
| PNW | ENA | 1.84 | 1.53 | 4 |

**Table S8**. Non-parametric ANOVAs (Kruskal-Wallis) testing for significant differences in number of eggs laid per female per day among parental and hybrid matings in crosses between pairwise combinations of ENA x SW, PNW x SW, and PNW x ENA flies.

| **Cross combination** | **Dependent** | **n** | **statistic** | **df** | **p** | **method** |
| --- | --- | --- | --- | --- | --- | --- |
|  |  |  |  |  |  |  |
| ENA_x_SW | Eggs per day | 99 | 4.53 | 3 | 0.21 | Kruskal-Wallis |
| PNW_x_SW | Eggs per day | 144 | 12.2 | 3 | 0.00688 | Kruskal-Wallis |
| PNW_x_ENA | Eggs per day | 57 | 11.6 | 3 | 0.00892 | Kruskal-Wallis |

**Table S9**. Non-parametric post-hoc test (Dunn’s Test) testing for differences in eggs laid per female per day between crosses involving ENA and SW flies, with the population origin for the female indicated first and male second for a given cross combination in Group 1 and Group 2 column.

| **group1** | **group2** | **n1** | **n2** | **statistic** | **p** | **p.adj** | **p.adj.signif** |
| --- | --- | --- | --- | --- | --- | --- | --- |
| PNW_x_PNW | PNW_x_SW | 35 | 18 | -0.27221278 | 0.785458415 | 1 | ns |
| PNW_x_PNW | SW_x_PNW | 35 | 31 | -2.166953239 | 0.03023842 | 0.148168257 | ns |
| PNW_x_PNW | SW_x_SW | 35 | 60 | -3.115152638 | 0.001838497 | 0.027025913 | * |
| PNW_x_SW | SW_x_PNW | 18 | 31 | -1.537102237 | 0.124268275 | 0.456685912 | ns |
| PNW_x_SW | SW_x_SW | 18 | 60 | -2.171656697 | 0.029881567 | 0.148168257 | ns |
| SW_x_PNW | SW_x_SW | 31 | 60 | -0.579232737 | 0.562432144 | 1 | ns |

**Table S10**. Non-parametric post-hoc Dunn’s tests for differences in eggs laid per female per day between among crosses involving ENA and PNW flies, with the population origin for the female indicated first and male second for a given cross combination in Group 1 and Group 2 columns.

| **group1** | **group2** | **n1** | **n2** | **statistic** | **p** | **p.adj** | **p.adj.signif** |
| --- | --- | --- | --- | --- | --- | --- | --- |
| ENA_x_ENA | ENA_x_PNW | 11 | 7 | 0.401884 | 0.68777 | 1 | ns |
| ENA_x_ENA | PNW_x_ENA | 11 | 4 | 0.600104 | 0.548437 | 1 | ns |
| ENA_x_ENA | PNW_x_PNW | 11 | 35 | 2.995407 | 0.002741 | 0.04029 | * |
| ENA_x_PNW | PNW_x_ENA | 7 | 4 | 0.249013 | 0.803351 | 1 | ns |
| ENA_x_PNW | PNW_x_PNW | 7 | 35 | 2.031408 | 0.042214 | 0.31027 | ns |
| PNW_x_ENA | PNW_x_PNW | 4 | 35 | 1.297854 | 0.194338 | 0.952254 | ns |

**Table S11.** Mean + s.e. egg hatch rate per cross for different mating combinations of PNW, SW, and ENA flies analyzed in the study. A total of 217 crosses from 2018 and 2019 were used to assess egg hatch rate, where n represents the number of different crosses in a given cross type.

| **♀ pop** | **♂Pop** | **Mean** | **s.e.** | **n** |
| --- | --- | --- | --- | --- |
| ENA | ENA | 0.39 | 0.09 | 11 |
| ENA | SW | 0.10 | 0.03 | 14 |
| SW | ENA | 0.42 | 0.09 | 9 |
| SW | SW | 0.31 | 0.04 | 51 |
| PNW | PNW | 0.53 | 0.04 | 54 |
| PNW | SW | 0.11 | 0.03 | 23 |
| SW | PNW | 0.36 | 0.06 | 25 |
| ENA | PNW | 0.57 | 0.07 | 14 |
| PNW | ENA | 0.37 | 0.09 | 16 |

**Table S12**. Non-parametric ANOVAs (Kruskal-Wallis) testing for significant differences in egg hatch rate among parental and hybrid cross types in mating between ENA x SW, PNW x SW, and PNW x ENA flies.

| **Cross combination** | **Dependent** | **n** | **statistic** | **df** | **p** | **method** |
| --- | --- | --- | --- | --- | --- | --- |
|  |  |  |  |  |  |  |
| ENA_x_SW | Hatch rate | 85 | 11.09098 | 3 | 0.0112 | Kruskal-Wallis |
| PNW_x_SW | Hatch rate | 153 | 34.06325 | 3 | 1.92E-07 | Kruskal-Wallis |
| PNW_x_ENA | Hatch rate | 95 | 5.551908 | 3 | 0.136 | Kruskal-Wallis |

**Table S13**. Non-parametric post-hoc Dunn’s tests for differences in egg hatch rate between different types of pairwise crosses involving ENA and SW flies, with the population origin for the female indicated first and male second for a given cross combination in Group 1 and Group 2 columns.

| **Group 1** | **Group 2** | **n1** | **n2** | **statistic** | **p** | **p.adj** | **p.adj.signif** |
| --- | --- | --- | --- | --- | --- | --- | --- |
| ENA_x_ENA | ENA_x_SW | 11 | 14 | -2.59224 | 0.009535 | 0.046723 | * |
| ENA_x_ENA | SW_x_ENA | 11 | 9 | 0.416607 | 0.676966 | 1 | ns |
| ENA_x_ENA | SW_x_SW | 11 | 51 | -0.70996 | 0.477728 | 1 | ns |
| ENA_x_SW | SW_x_ENA | 14 | 9 | 2.882866 | 0.003941 | 0.046723 | * |
| ENA_x_SW | SW_x_SW | 14 | 51 | 2.679361 | 0.007376 | 0.046723 | * |
| SW_x_ENA | SW_x_SW | 9 | 51 | -1.17071 | 0.241715 | 0.888303 | ns |

**Table S14**. Non-parametric post-hoc Dunn’s tests for differences in egg hatch rate between different types of pairwise crosses involving PNW and SW flies, with the population origin for the female indicated first and male second for a given cross combination in Group 1 and Group 2 columns.

| **Group 1** | **Group 2** | **n1** | **n2** | **statistic** | **p** | **p.adj** | **p.adj.signif** |
| --- | --- | --- | --- | --- | --- | --- | --- |
| PNW_x_PNW | PNW_x_SW | 54 | 23 | -5.67686 | 1.37E-08 | 2.02E-07 | **** |
| PNW_x_PNW | SW_x_PNW | 54 | 25 | -2.1214 | 0.033888 | 0.099631 | ns |
| PNW_x_PNW | SW_x_SW | 54 | 51 | -3.41502 | 0.000638 | 0.004688 | ** |
| PNW_x_SW | SW_x_PNW | 23 | 25 | 3.116061 | 0.001833 | 0.008981 | ** |
| PNW_x_SW | SW_x_SW | 23 | 51 | 2.972788 | 0.002951 | 0.010845 | * |
| SW_x_PNW | SW_x_SW | 25 | 51 | -0.62928 | 0.529165 | 1 | ns |

**Table S15**. *Wolbachia* infection status of flies in North American cherry fly populations based on TEEseq of six MLST loci for stains *w*Cin2 and *w*Cin3. Site numbers correspond to those in Table S1, and Figure 5.

| **Site number** | **pop** | **Locality** | **n** | ***w*Cin2** | ***w*Cin2 & 3** | | ***w*Cin3** |
| --- | --- | --- | --- | --- | --- | --- | --- |
| 1 | PNW | Vancouver, WA | 7 | 7 | 0 | 0 | |
| 3 | PNW | Aplin Rd., White Salmon, WA | 8 | 8 | 0 | 0 | |
| 4 | PNW | Nile, WA | 5 | 5 | 0 | 0 | |
| 5 | PNW | Salmon Arm, BC, Can | 8 | 8 | 0 | 0 | |
| 6 | PNW | Kennewick, WA | 5 | 5 | 0 | 0 | |
| 7 | PNW | Rose Lake, ID | 8 | 8 | 0 | 0 | |
| 8 | PNW | Somers, MT | 5 | 5 | 0 | 0 | |
| 10 | SW | Chriacahoua Mtns, AZ | 7 | 0 | 7 | 0 | |
| 11 | SW | Gila Cliff Dwelling Mon., NM | 8 | 1 | 7 | 0 | |
| 12 | SW | Davis Mountains, TX | 7 | 0 | 7 | 0 | |
| 13 | SMO | Nuevo León, Mex | 8 | 1 | 7 | 0 | |
| 14 | SMO | Los Lirios, Coahuila, Mex | 7 | 0 | 7 | 0 | |
| 15 | EVTM | Tlalpan, Distrito Federal (CDMX), Mex | 6 | 0 | 6 | 0 | |
| 16 | EVTM | Tulancingo, Hidalgo, Mex | 7 | 6 | 1 | 0 | |
| 17 | EVTM | Huamantla, Tlaxcala, Mex | 6 | 0 | 1 | 5 | |
| 18 | ENA | Scott Park, Iowa City, IO | 8 | 8 | 0 | 0 | |
| 19 | ENA | The Dells, Wisconsin Dells, WI | 6 | 6 | 0 | 0 | |
| 20 | ENA | Lake of the Woods, IL | 8 | 8 | 0 | 0 | |
| 21 | ENA | South Bend, IN | 7 | 7 | 0 | 0 | |
| 22 | ENA | Live Oak, Suwannee County, FL | 7 | 7 | 0 | 0 | |
| 23 | ENA | Gainesville, FL-Health, FL | 7 | 7 | 0 | 0 | |
| 24 | ENA | Green Creek, Cape May, NJ | 7 | 7 | 0 | 0 | |

**Table S16**. Sanger sequencing results to test that *Wolbachia* infection status and mtDNA haplotypes of individual cherry flies used in crossing experiments correspond to populations surveys based on TEEseq and the findings of Doellman et al. (2019).

| **FLYD** | **pop** | **HCPA_genotype** | ***wsp*_genotype** | **COI_genotype** |
| --- | --- | --- | --- | --- |
| 19DB073 | ENA | *w*Cin2 | *w*Cin2 | PNW/ENA |
| 19DB074 | ENA | *w*Cin2 | *w*Cin2 | PNW/ENA |
| 19DB050 | ENA | *w*Cin2 | *w*Cin2 | PNW/ENA |
| 19DB299.1 | ENA | *w*Cin2 | *w*Cin2 | PNW/ENA |
| 19DB299.2 | ENA | *w*Cin2 | *w*Cin2 | PNW/ENA |
| DB18.0465 | ENA | *w*Cin2 | *w*Cin2 | PNW/ENA |
| DB18.0464 | ENA | *w*Cin2 | *w*Cin2 | PNW/ENA |
| DB18.0537 | ENA | *w*Cin2 | *w*Cin2 | PNW/ENA |
| DB18.0490.1 | ENA | *w*Cin2 | *w*Cin2 | PNW/ENA |
| DB18.0490.2 | ENA | *w*Cin2 | *w*Cin2 | PNW/ENA |
| 19DB511 | ENA | *w*Cin2 | *w*Cin2 | PNW/ENA |
| 19DB575 | ENA | *w*Cin2 | *w*Cin2 | PNW/ENA |
| DB18.0620 | ENA | *w*Cin2 | *w*Cin2 | PNW/ENA |
| 19DB418 | ENA | *w*Cin2 | *w*Cin2 | PNW/ENA |
| 19DB419 | ENA | *w*Cin2 | *w*Cin2 | PNW/ENA |
| 19DB505 | ENA | *w*Cin2 | *w*Cin2 | PNW/ENA |
| 19DB313 | ENA | *w*Cin2 | *w*Cin2 | PNW/ENA |
| 19DB366 | ENA | *w*Cin2 | *w*Cin2 | PNW/ENA |
| 19DB042 | PNW | *w*Cin2 | *w*Cin2 | PNW/ENA |
| 19DB046 | PNW | *w*Cin2 | *w*Cin2 | PNW/ENA |
| 19DB047 | PNW | *w*Cin2 | *w*Cin2 | PNW/ENA |
| 19DB048 | PNW | *w*Cin2 | *w*Cin2 | PNW/ENA |
| DB18.0369.2 | PNW | *w*Cin2 | *w*Cin2 | PNW/ENA |
| DB18.0468 | PNW | *w*Cin2 | *w*Cin2 | PNW/ENA |
| DB18.0614 | PNW | *w*Cin2 | *w*Cin2 | PNW/ENA |
| DB18.0618 | PNW | *w*Cin2 | *w*Cin2 | PNW/ENA |
| DB18.0647 | PNW | *w*Cin2 | *w*Cin2 | PNW/ENA |
| 19DB512 | PNW | *w*Cin2 | *w*Cin2 | PNW/ENA |
| 19DB365 | PNW | *w*Cin2 | *w*Cin2 | PNW/ENA |
| DB18.0215 | PNW | *w*Cin2 | *w*Cin2 | PNW/ENA |
| DB18.0402 | PNW | *w*Cin2 | *w*Cin2 | PNW/ENA |
| 19DB627 | PNW | *w*Cin2 | *w*Cin2 | PNW/ENA |
| 19DB406 | PNW | *w*Cin2 | *w*Cin2 | PNW/ENA |
| 19DB152A | SW | *w*Cin2+*w*Cin3 | *w*Cin2+*w*Cin3 | SW |
| 19DB152B | SW | *w*Cin2+*w*Cin3 | *w*Cin2+*w*Cin3 | SW |
| 19DB152C | SW | *w*Cin2+*w*Cin3 | *w*Cin2+*w*Cin3 | SW |
| 19DB152D | SW | *w*Cin2+*w*Cin3 | *w*Cin2+*w*Cin3 | SW |
| 19DB152E | SW | *w*Cin2+*w*Cin3 | *w*Cin2+*w*Cin3 | SW |
| DB18.0466 | SW | *w*Cin2+*w*Cin3 | *w*Cin2+*w*Cin3 | SW |
| DB18.0461_re | SW | *w*Cin2+*w*Cin3 | *w*Cin2+*w*Cin3 | SW |
| DB18.0525 | SW | *w*Cin2 | *w*Cin2+*w*Cin3 | SW |
| DB18.0379A | SW | *w*Cin2+*w*Cin3 | *w*Cin2+*w*Cin3 | SW |
| DB18.0379B | SW | *w*Cin2+*w*Cin3 | *w*Cin2+*w*Cin3 | SW |
| 19DB140_I | SW | *w*Cin2+*w*Cin3 | *w*Cin2+*w*Cin3 | SW |
| 19DB140_F | SW | *w*Cin2+*w*Cin3 | *w*Cin2+*w*Cin3 | SW |
| 19DB140_C | SW | *w*Cin2+*w*Cin3 | *w*Cin2+*w*Cin3 | SW |
| 19DB140_G | SW | *w*Cin2+*w*Cin3 | *w*Cin2+*w*Cin3 | SW |
| 19DB140_E | SW | *w*Cin2+*w*Cin3 | *w*Cin2+*w*Cin3 | SW |
| DB18.0523 | SW | *w*Cin2+*w*Cin3 | *w*Cin2+*w*Cin3 | SW |
| 19DB355B | SW | *w*Cin2+*w*Cin3 | *w*Cin2+*w*Cin3 | SW |
| 19DB355C | SW | *w*Cin2+*w*Cin3 | *w*Cin2+*w*Cin3 | SW |
| 19DB355D | SW | *w*Cin2+*w*Cin3 | *w*Cin2+*w*Cin3 | SW |
| 19DB355E | SW | *w*Cin2+*w*Cin3 | *w*Cin2+*w*Cin3 | SW |
| 19DB355F | SW | *w*Cin2+*w*Cin3 | *w*Cin2+*w*Cin3 | SW |
| DB18.0384.3 | SW | *w*Cin2+*w*Cin3 | *w*Cin2+*w*Cin3 | SW |
| DB18.0373 | SW | *w*Cin2+*w*Cin3 | *w*Cin2+*w*Cin3 | SW |
| DB18.0467 | SW | *w*Cin2+*w*Cin3 | *w*Cin2+*w*Cin3 | SW |
| DB18.0145 | SW | *w*Cin2+*w*Cin3 | *w*Cin2+*w*Cin3 | SW |
| DB18.0685 | SW | *w*Cin2+*w*Cin3 | *w*Cin2+*w*Cin3 | SW |
| DB18.0615 | SW | *w*Cin2+*w*Cin3 | *w*Cin2+*w*Cin3 | SW |
| 19DB576 | SW | *w*Cin2+*w*Cin3 | *w*Cin2+*w*Cin3 | SW |
| DB18.0619 | SW | *w*Cin2+*w*Cin3 | *w*Cin2+*w*Cin3 | SW |
| 19DB509 | SW | *w*Cin2+*w*Cin3 | *w*Cin2+*w*Cin3 | SW |
| 19DB510 | SW | *w*Cin2+*w*Cin3 | *w*Cin2+*w*Cin3 | SW |
| 19DB506 | SW | *w*Cin2+*w*Cin3 | *w*Cin2+*w*Cin3 | SW |
| DB18.0214 | SW | *w*Cin2+*w*Cin3 | *w*Cin2+*w*Cin3 | SW |
| DB18.0401 | SW | *w*Cin2+*w*Cin3 | *w*Cin2+*w*Cin3 | SW |
| 19DB628 | SW | *w*Cin2+*w*Cin3 | *w*Cin2+*w*Cin3 | SW |
| 19DB422 | SW | *w*Cin2+*w*Cin3 | *w*Cin2+*w*Cin3 | SW |
| 19DB405 | SW | *w*Cin2+*w*Cin3 | *w*Cin2+*w*Cin3 | SW |
| DB18.0609 | SW | NA | *w*Cin2+*w*Cin3 | SW |

**Table S17**. Mean proportion of *w*Cin3 TEEseq reads for individuals coinfected with *w*Cin2 and *w*Cin3 from coinfected populations in the SW, SMO, and EVTM.

| **site** | **pop** | ***coxA*** | ***wsp*** | ***ftsZ*** | ***fbpA*** | ***hcpA*** | ***gatB*** | **mean** |
| --- | --- | --- | --- | --- | --- | --- | --- | --- |
| 10 | SW | 0.033 | 0.034 | 0.033 | 0.023 | 0.050 | 0.027 | 0.032 |
| 11 | SW | 0.147 | 0.199 | 0.147 | 0.090 | 0.239 | 0.094 | 0.143 |
| 12 | SW | 0.075 | 0.091 | 0.075 | 0.057 | 0.104 | 0.055 | 0.074 |
| 13 | SMO | 0.172 | 0.210 | 0.172 | 0.087 | 0.241 | 0.080 | 0.148 |
| 14 | SMO | 0.415 | 0.367 | 0.415 | 0.216 | 0.424 | 0.160 | 0.312 |
| 15 | EVTM | 0.112 | 0.088 | 0.112 | 0.037 | 0.080 | 0.020 | 0.064 |

**Table S18.** Probabilities of gene flow *P*(*GH*) calculated from levels of allochronic, sexual, and post-mating isolation measured here and in the study of Tadeo et al. (2015) and critical migration rates (*m*_k_) given uni- or bi-directional cytoplasmic incompatibility (CI) estimated for varying transmission rates (t) between pairs of PNW, SW, ENA, SMO, and EVTM populations. When *P*(*GH*) < *m*_k_ it is possible for coupling of CI and other forms of non-endosymbiont forms of RI to impede the spread of *Wolbachia* between cherry fly populations if they were to come into secondary contact.

|  |  | Premating | | Post-mating | **Total non-endosymbiont** | **Endosymbiont-related** |  |  |
| --- | --- | --- | --- | --- | --- | --- | --- | --- |
| Pop. 1 | Pop. 2 | Allochronic | Sexual | Egg lay | ***P(GH)*** | Cyto. Incompat. (CI) | *t* | ***m*_k_** |
|  |  |  |  |  |  |  |  |  |
| SW | PNW | 15-d: 0.00215 | 0.295 | 1.000 | **6.342 x 10-4** | 0.800 unidirectional | 0.990 | **3.189 x 10-5** |
|  |  | 30-d: 0.01750 | 0.295 | 1.000 | **5.163 x 10-3** |  | 0.950 | **8.679 x 10-4** |
|  |  |  |  |  |  |  | 0.900 | **3.907 x 10-3** |
|  |  |  |  |  |  |  | 0.875 | **6.519 x 10-3** |
|  |  |  |  |  |  |  |  |  |
| SW | ENA | 15-d: 0.00523 | 0.307 | 1.000 | **1.612 x 10-3** | 0.738 unidirectional | 0.990 | **3.464 x 10-5** |
|  |  | 30-d: 0.06730 | 0.307 | 1.000 | **2.066 x 10-2** |  | 0.950 | **9.525 x 10-4** |
|  |  |  |  |  |  |  | 0.900 | **4.364 x 10-3** |
|  |  |  |  |  |  |  | 0.875 | **7.372 x 10-3** |
|  |  |  |  |  |  |  |  |  |
| SMO | ENA | 15-d: 0.00165 | 0.300† | 0.129 | **6.386 x 10-5** | 0.907 unidirectional | 0.990 | **2.804 x 10-5** |
|  |  | 30-d: 0.01435 | 0.300† | 0.129 | **5.553 x 10-4** |  | 0.950 | **7.527 x 10-4** |
|  |  |  |  |  |  |  | 0.900 | **3.312 x 10-3** |
|  |  |  |  |  |  |  | 0.875 | **5.444 x 10-3** |
|  |  |  |  |  |  |  |  |  |
| SMO | PNW | 15-d: 0.00055 | 0.300† | 0.129‡ | **2.129 x 10-5** | 0.907‡ unidirectional | 0.990 | **2.804 x 10-5** |
|  |  | 30-d: 0.00650 | 0.300† | 0.129‡ | **2.516 x 10-4** |  | 0.950 | **7.527 x 10-4** |
|  |  |  |  |  |  |  | 0.900 | **3.312 x 10-3** |
|  |  |  |  |  |  |  | 0.875 | **5.444 x 10-3** |
|  |  |  |  |  |  |  |  |  |
| EVTM | ENA | 15-d: 0.14215 | 0.300† | 0.115 | **4.904 x 10-3** | 0.907§ bidirectional | -- | **1.205 x 10-1** |
|  |  | 30-d: 0.28275 | 0.300† | 0.115 | **9.755 x 10-3** | 0.797§ |  |  |
|  |  |  |  |  |  |  |  |  |
| EVTM | PNW | 15-d: 0.03675 | 0.300† | 0.115‡ | **1.268 x 10-3** | 0.907‡ bidirectional | **--** | **1.205 x 10-1** |
|  |  | 30-d: 0.11205 | 0.300† | 0.115‡ | **3.866 x 10-3** | 0.797‡ |  |  |
|  |  |  |  |  |  |  |  |  |
| EVTM | SMO | 15-d: 0.05055 | 0.300 | 1.000 | **1.517 x 10-3** | 0.797 unidirectional | 0.990 | **3.201 x 10-5** |
|  |  | 30-d: 0.08715 | 0.300 | 1.000 | **2.615 x 10-3** |  | 0.950 | **8.716 x 10-4** |
|  |  |  |  |  |  |  | 0.900 | **3.927 x 10-3** |
|  |  |  |  |  |  |  | 0.875 | **6.556 x 10-3** |

† Estimates of sexual isolation between EVTM and SMO used for EVTM comparisons to USA, as Tadeo et. al. (2015) did not perform crosses between these flies. Value of 0.300 represents conservative estimate corresponding to levels of reduction in gene flow seen between other regional comparisons of cherry fly populations.

‡ Estimates of reduction in egg laying and egg hatch (CI) between ENA and Mexican flies used for PNW comparisons to Mexico, as Tadeo et. al. (2015) did not perform crosses between these flies.

§ Estimate of the effect of *w*Cin3 on CI based on reduction in egg hatch rate from Tadeo et al. (2015) in crosses between SMO males with strain and ENA lacking the strain. Estimate of the effect of *w*Cin2 on CI based on reduction in egg hatch rate from Tadeo et al. (2015) in crosses between SMO females with the strain and EVTM males generally lacking the strain.

**Supplementary References**

Doellman, M. M., Schuler, H., Jean, G. S., Hood, G. R., Egan, S. P., Powell, T. H. Q., … Feder, J. L. (2019). Geographic and ecological dimensions of host plant-associated genetic differentiation and speciation in the *Rhagoletis cingulata* (Diptera: Tephritidae) sibling species group. *Insects*, *10*(9), 275. doi: 10.3390/insects10090275

Feder, J. L., Hunt, T. A., & Bush, L. (1993). The effects of climate, host plant phenology and host fidelity on the genetics of apple and hawthorn infesting races of *Rhagoletis pomonella*. *Entomologia Experimentalis et Applicata*, *69*(2), 117–135. doi: https://doi.org/10.1111/j.1570-7458.1993.tb01735.x

Tadeo, E., Feder, J. L., Egan, S. P., Schuler, H., Aluja, M., & Rull, J. (2015). Divergence and evolution of reproductive barriers among three allopatric populations of *Rhagoletis cingulata* across eastern North America and Mexico. *Entomologia Experimentalis et Applicata*, *156*(3), 301–311. doi: 10.1111/eea.12331
